# Improved electroless platinum contacts on CdZnTe X- and γ-rays detectors

**DOI:** 10.1038/s41598-020-70801-9

**Published:** 2020-08-13

**Authors:** Manuele Bettelli, Nicola Sarzi Amadè, Silvia Zanettini, Lucia Nasi, Marco Villani, Leonardo Abbene, Fabio Principato, Andrea Santi, Maura Pavesi, Andrea Zappettini

**Affiliations:** 1 IMEM-CNR, 43124 Parma, Italy; 2 Due2lab s.r.l., 43121 Parma, Italy; 3 Department of Physics and Chemistry (DiFC)-Emilio Segrè, University of Palermo, 90128 Palermo, Italy; 4 Department of Mathematical, Physical and Computer Sciences, University of Parma, 43124 Parma, Italy

**Keywords:** Materials science, Materials for devices

## Abstract

Platinum is a promising candidate for the realization of blocking electrical contacts on cadmium-zinc-telluride (CdZnTe or CZT) radiation detectors. However, the poor mechanical adhesion of this metal often shortens the lifetime of the final device. In this work, a simple and effective procedure to obtain robust platinum contacts by electroless deposition is presented. Microscopical analysis revealed the final thickness and composition of the contact layer and its adhesion to the bulk crystal. The blocking nature of the Pt-CdZnTe junction, essential to obtain low noise devices, was confirmed by current–voltage measurements. The planar Pt-CdZnTe-Pt detectors showed good room temperature spectroscopic performance with energy resolution of 4% (2.4 keV) and 3% (3.7 keV) FWHM at 59.5 and 122.1 keV, respectively. Finally, we showed, for the first time, that platinum contacts allow the estimation of the carrier lifetime and mobility of both holes and electrons by using current transient measurements. This demonstrated the optimal hole extraction capability of such contacts.

## Introduction

CdTe and CdZnTe (CZT) have proven to be excellent materials for room temperature X-ray and gamma-ray detectors and they are gradually replacing other sensing systems in many applications. Electrical contacts on such detectors need to be customised according to the final purpose of the device and engineered depending on the properties of the starting material such as work function and bulk resistivity. A great interest has been addressed to platinum metal contacts^1–3^ because Pt work function (~ 5.6 eV) leads to the formation of a high Pt–CZT Schottky barrier which limits the carrier injection and thus the leakage current. This is the reason why blocking contacts are usually adopted to realize high-resolution spectrometers^4^.

Electroless deposition technique is vastly used to deposit contacts on II/VI materials. The reason lies in its simplicity since no specific or expensive laboratory equipment is required. This method consists in the spontaneous reaction of the metal in salt form dissolved in a proper solvent with the detector material. The reaction takes place without the use of any external electric power and allows to realize chemically stable and reproducible contacts. Moreover, the oxide formation at the metal/semiconductor interface is minimised since contacts are realised immediately after the polishing step, hence reducing the air exposition of the highly reactive surface. This is crucial to reduce the effect of the oxide layer on the leakage current and charge collection efficiency. Lastly, fine tuning of electroless deposition parameters allows to control the final quality of the metallic layer in term of thickness and homogeneity.

In this work, Pt contacts have been deposited by means of a electroless deposition technique based on alcoholic solution. The CdZnTe detectors have been realised and characterised thorough mechanical, structural, electrical, and spectroscopic analysis. This study is focused on explaining the role of the metal/semiconductor junction on the charge collection efficiency for both electrons and holes and, thus, on the detector final performance. Results are compared to standard Au electroless contacts.

## Methods

### Sample preparation

Pt and Au contacts were deposited both on CZT samples grown by Traveller Heating Method purchased from REDLEN Technologies (Canada) and on CZT samples cut from crystals grown by the Boron Oxide Encapsulated Vertical Bridgman Technique at IMEM-CNR^5^. In the latter case, the polycrystalline charge was obtained by direct synthesis of the pure elements^6^ and thermally treated before growth in such a way to reduce the off-stoichiometry^7^. Before platinum deposition, surfaces were lapped by means of abrasive paper with particle size of 8 and 5 μm and then polished with 3 and 1 μm diamond powders. In order to minimize surface oxidation, metal contacts were deposited onto the sample surface immediately after the polishing step. Gold was deposited by using electroless deposition using alcoholic solutions^8^ whereas platinum was deposited by using the technique described below. In order to verify the mechanical resistance of contacts, we performed the tape test using a standard tape (model 3M 600) and following the ISO 2409 procedure which allows to compare results from different deposition techniques^9,10^. Lastly, we performed the photolithography of the samples by etching the metal contact with a Br_2_ solution.

### Structural characterization

Cross-sectional Transmission Electron Microscopy (TEM) and Energy Dispersive X-ray Spectroscopy (EDX) have been performed in order to investigate the structural and material properties of the Pt layer. EDX maps have been obtained in Scanning-TEM (STEM) mode using high-angle annular dark-field (HAADF) detector.

For current–voltage characterization and spectroscopic characterization, a 5.5 × 5.5 × 2 mm^3^ sample (sample AA) and a 6 × 5.7 × 1.1 mm^3^ sample (sample PP) were cut from the same REDLEN detector. A full-area cathode and a 4 × 4 mm^2^ anode (pixel), this last surrounded by a guard ring, were patterned by means of photolithography on the opposite faces of both samples (see Supplementary Fig. S1 online). Current/voltage characteristics were performed with a Keithley 2410 sourcemeter and a Keithley 6485 picoammeter.

### X-ray and gamma ray measurements

Uncollimated X-ray and gamma ray calibration sources (gamma-ray lines: ^241^Am, 59.5 and 26.3 keV; ^57^Co, 122.1 and 136.5 keV) were used to characterize the spectroscopic performance of the detectors. The 14 keV gamma line of the ^57^Co source is shielded by the source holder. Tungsten K X-ray fluorescent lines (K_α1_ = 59.3 keV, K_α2_ = 58.0 keV, K_β1_ = 67.2 keV, K_β3_ = 66.9 keV) and Np L X-ray lines are also emitted by ^57^Co and ^241^Am sources, respectively. All detectors were irradiated through the cathode side. The detectors signals were amplified by using a resistive feedback ac-coupled charge sensitive preamplifier CSP (A250, Amptek, USA) with a nominal equivalent noise charge (ENC) of 100 electrons. Energy X-ray and gamma ray spectra were obtained with a standard analog pulse processing electronics: shaping amplifier (672, ORTEC, USA) with a shaping time constant of 1 μs and multichannel analyser (MCA-8000A, Amptek, USA). Further details of spectroscopic set-up are reported in previous works^11^.

### Laser induced transient current measurements

As a further investigation, carrier mobility and lifetime measurements were obtained by means of Laser Induced Transient Current Technique (LI-TCT)^12^ on an additional set of samples based on CZT from REDLEN and from IMEM-CNR, on which Pt contacts were deposited on the opposite surfaces.

Two samples with platinum contacts were manufactured for laser-induced transient current characterization. The first sample (A) was cut from a crystal grown at IMEM by Boron Encapsulated Vertical Bridgman technique, and had dimensions of 4 × 4 × 1 mm^3^. A central 2 × 2 mm^2^ pixel surrounded by the guard ring was realized on the cathode. The second sample (B) was obtained by a REDLEN detector and had dimensions of 6 × 6 × 1.1 mm^3^. As anode contact a central 4 × 4 mm^2^ pixel surrounded by the guard ring was realized (see Supplementary Fig. S2 online). In order to prevent undesired interaction with light during the measurements, samples were completely encapsulated in epoxy glue except for a narrow hole through which the sample was irradiated by means of an optical fiber. The typical applied bias voltage varied greatly depending on the type of investigated carrier because of the difference between electron and hole ${\upmu \uptau }$ product in CZT: from few to tens of volts in the former case; from tens to hundreds of volts in the latter.

Making use of a Nd:YAG Polaris II laser system, samples have been optically excited impinging an area of about few hundreds of μm in diameter, at the center of the cathode. 532 nm pulses (second harmonic of Nd:YAG laser) are 10 ns long, and with maximum repetition rate of 20 Hz. Light is simultaneously brought on the sample and on a fast photodiode that acts as pulse trigger by means of two optical fibers of the same length. The pulse energy released on the CZT is the same for all the acquired current transients. Low power laser pulse has been used in order to prevent transient distortions due to high number of photogenerated charges. To reduce noise contribution and little fluctuation of photogenerated charge, a few thousands of current pulses have been averaged thanks to the trigger system.

The output signals, coming from the pixel, have been amplified by a home-made amplifier (up to 400 times) with bandwidth of 80 MHz. Bias voltage has been supplied by a Keithley 2410 High Voltage Source Measure Unit. The current transients have been acquired by a DS8202 Owon digital oscilloscope with 1 GHz bandwidth and saved on a computer by means of Matlab software.

By using an excitation energy above the energy gap and changing the sign of bias, LI-TCT measurements allow to study transport features for one kind of charge carrier at a time as a consequence of the Ramo-Shockley theorem^13^. The attenuation length of a tenth of a μm at 532 nm, as estimated by the absorption coefficient of Cd_0.9_Zn_0.1_Te reported in^14^, ensures photon absorption takes place mainly at a depth, underneath the illuminated contact, that is only about 0.02% of the active thickness of detectors. It is then correct to assume the main amount of charges travelling inside the detector are generated immediately under the illuminated contact.

The electrodes are thin enough to ensure that the majority of the incoming photons reaches the active region of the detectors. The lateral surface of samples has been shielded to prevent absorption of reflected/diffused light; this is important to prevent unwanted generation of signal at depths other than that just below the metal contact where the laser is focused.

Experiments here reported require a low repetition rate to ensure the laser does not affect the polarization of material. Repetition rate and beam energy are carefully calibrated to ensure a negligible variation of space charge inside the samples.

LI-TCT method, used in this work and described in^12^ or more in detail in^15^, could be useful to investigate space charge distribution inside the volume of detectors even after high flux damaging. For a comparison with other approaches, see also^16–18^.

### Contact deposition

Firstly, Pt contacts were realized using standard electroless procedures^1,2^. The electroless solutions were prepared with platinum chloride (PtCl_4_) as precursor which can react both with Cd and Te^2^:

$$PtC{l}_{6}^{2-}+C{d}_{\left(s\right)}\stackrel{ }{\Rightarrow }PtC{l}_{4}^{2-}+2C{l}^{-}+C{d}^{2+}\gg\Delta E=+1.09V$$

$$PtC{l}_{4}^{2-}+C{d}_{\left(s\right)}\stackrel{ }{\Rightarrow }P{t}_{\left(s\right)}+4C{l}^{-}+C{d}^{2+}\gg\Delta E=+1.16V$$

$$2PtC{l}_{6}^{2-}+T{e}_{\left(s\right)}\stackrel{ }{\Rightarrow }2PtC{l}_{4}^{2-}+4C{l}^{-}+T{e}^{4+}\gg\Delta E=+0.11V$$

$$2PtC{l}_{4}^{2-}+T{e}_{\left(s\right)}\stackrel{ }{\Rightarrow }P{t}_{\left(s\right)}+8C{l}^{-}+T{e}^{4+}\gg\Delta E=+0.19V$$

**Figure 1:**
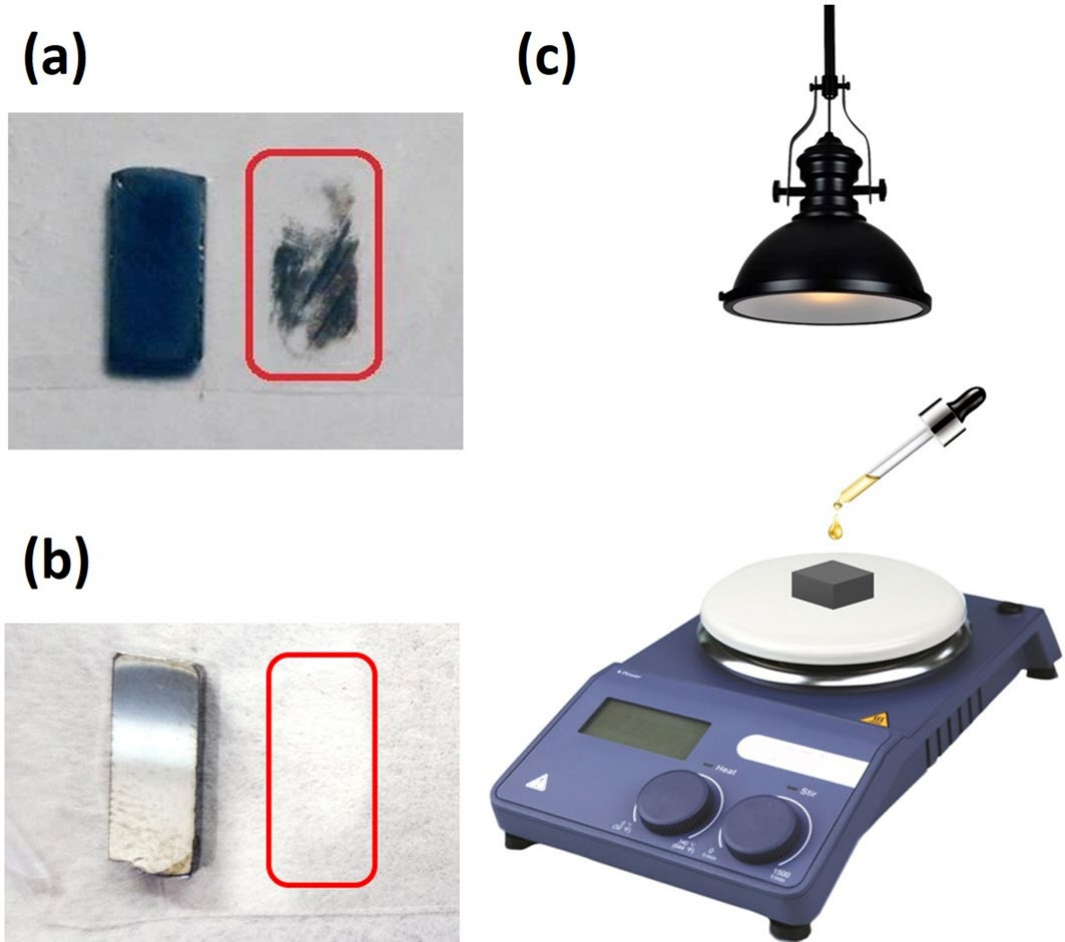
Platinum contact obtained by standard electroless procedure and the tape status after the tape test (**a**); Platinum contact obtained by MeOH/DMF electroless procedure, and the tape status after the tape test (**b**); Schematic representation of the Pt deposition setup (**c**).

The different chemical potentials indicate that platinum reacts mainly with cadmium, thus transferring Cd^2+^ ions and leaving a Te-rich layer on CZT on which metal Pt atoms precipitate^8,19^. Thus far, platinum deposition was not an easy and always successful process. Literature reports platinum electroless deposition from water or methanol solutions at room temperature^1,2^. However, platinum layers deposited in such conditions (2% of PtCl_4_ in water solution at 25 °C) are not uniform and consist of islands with various extent and thickness, even if salt concentration and deposition time are varied. These contacts also show poor mechanical stability and cannot withstand the tape test (Fig. 1a). In fact, adhesion is still one of the major issues for contacts on CZT, the situation being worsened by the instability of CZT at high temperature that cause the impossibility of annealing contacts.

By using a 4% (w/w) platinum chloride solution in 1:1 methanol/dimethylformamide (DMF), an optimal deposition of Pt film can be achieved. Heating the solution up to 60–70 °C with mechanical stirring significantly speeds up the dissolution of platinum salt. The resulting electroless solution can be stored in an amber vial at 4 °C for several weeks. DMF has been previously investigated as a solvent for the synthesis of semiconducting^20^ and metal nanoparticles (NPs)^21^ mainly because of the high dielectric constant and coordinating ability. DMF can reduce metal salts and form NPs or films depending on the synthetic condition (mainly solution supersaturation and ligand complexation), even though the room temperature reaction, without reducing agents, can be very slow^22^. In the proposed method, samples were placed on a hotplate heated up to 100 °C, then 50 μL of the cooled (4 °C) solution was drop-casted on the samples and illuminated with a 300 W halogen lamp for 5 min (Fig. 1c). This triggers the electroless deposition of the platinum layer. The drop-casting procedure can be repeated several times to tailor the Pt layer thickness. We found that repeating 3 times ensures a uniform, 60 nm thick, film deposition. Finally, samples were cooled down and rinsed with deionized water.

The final metal layer appears homogeneous, shiny and smooth (Fig. 1b). After this procedure, Pt contacts withstand at last the tape test.

## Results

### TEM analyses

**Figure 2:**
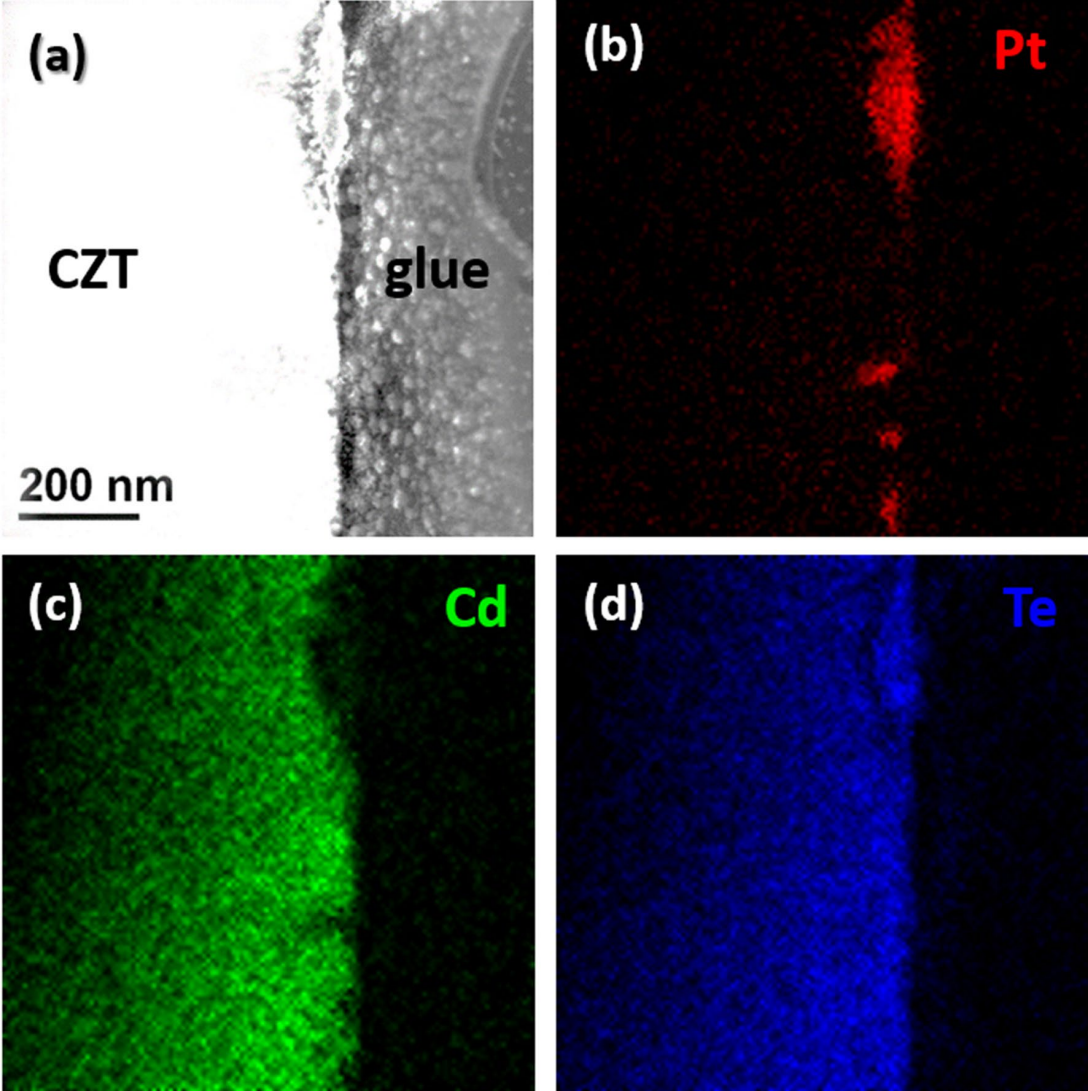
Cross section TEM–EDX analysis for the Pt contact obtained in water, STEM-HAADF image (**a**) and corresponding EDX maps for Pt (**b**), Cd (**c**), Te (**d**).

Figure 2 shows the cross section TEM–EDX analysis for the Pt contact obtained in water, the STEM-HAADF image (a) and corresponding EDX maps for Pt (b), Cd (c), Te (d). Images reveal that the contact is formed by Pt islands and is, thus, very inhomogeneous.

**Figure 3:**
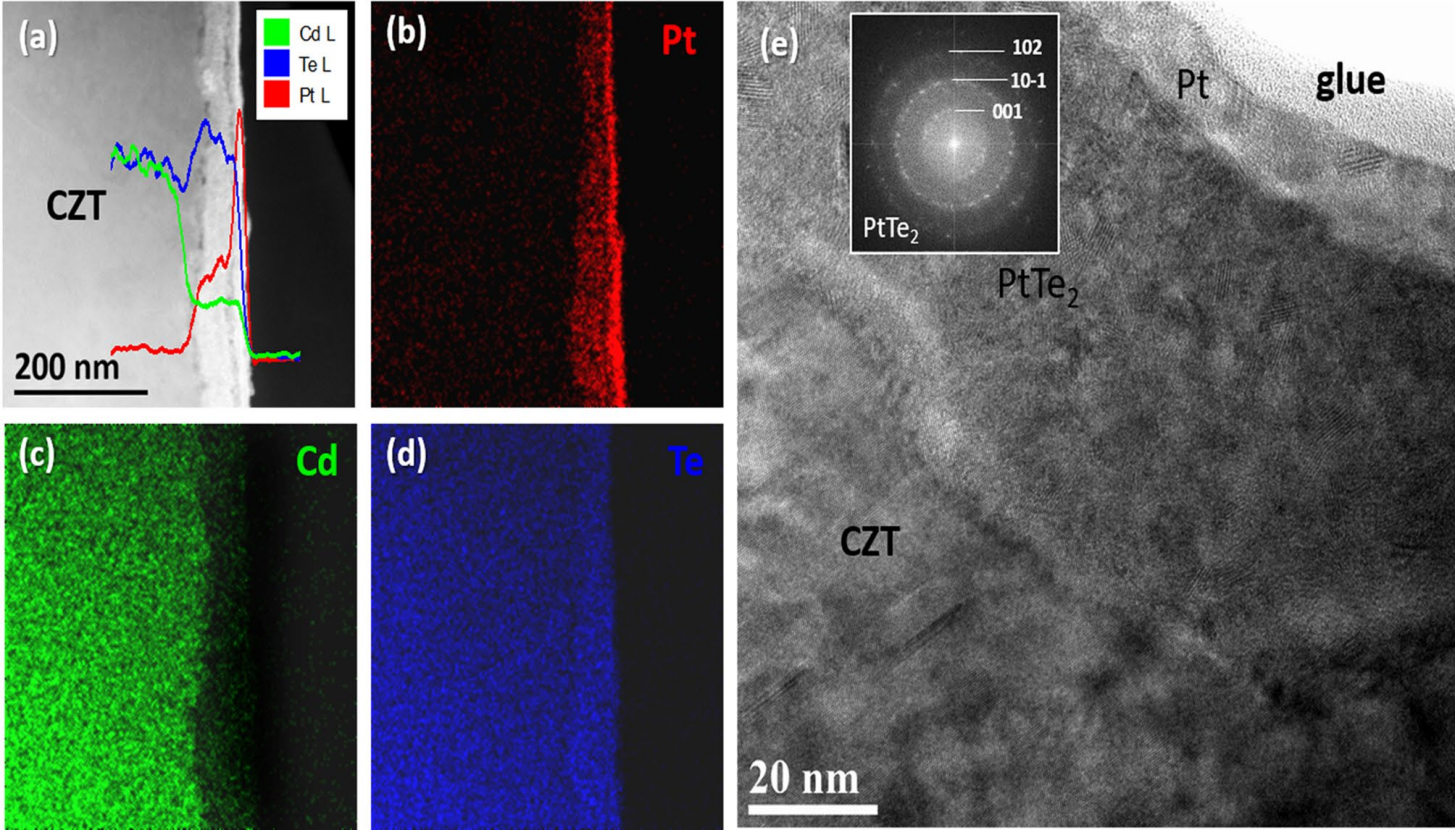
(**a**) Cross section STEM-HAADF image with the superimposed composition profile of Pt, Cd and Te. (**b**–**d**) Pt, Cd and Te EDX maps of the same region. (**e**) High Resolution TEM of the MeOH/DMF Pt contact with the Fast Fourier transform of the PtTe_2_ layer in the inset.

Figure 3a shows a STEM-HAADF image of the MeOH/DMF Pt contact and the relative composition profile of Pt, Cd and Te. EDX maps of the same region are reported in Fig. 3b–d. The thickness of the deposited contact layer is about 60 nm and is continuous in respect to that obtained by deposition in water. As revealed by the composition profile, the contact layer is composed of a top 20 nm-thick Pt layer, followed by a region of ∽ 40 nm characterized by a mixture of Pt and Te. In correspondence with this layer, a Cd depleted region can be noticed which is typical of electroless deposition^19,23^. The extent of the Cd depletion has been previously associated to the mechanical stability of the contact^8^. In the present case, this does not hold, because a Pt-Te compound is formed.

In order to identify the phase of the PtTe layer, High Resolution TEM was employed in this region (Fig. 3e). The presence of rings in the Fast Fourier Transform pattern confirms the polycrystallinity of the contact layer and their position is consistent with that of a PtTe_2_ alloy. This explains also the increase of Te contents in respect to the bulk material (Fig. 3a). PtTe_2_ is known to be metallic alloy.

### Electrical properties

**Figure 4:**
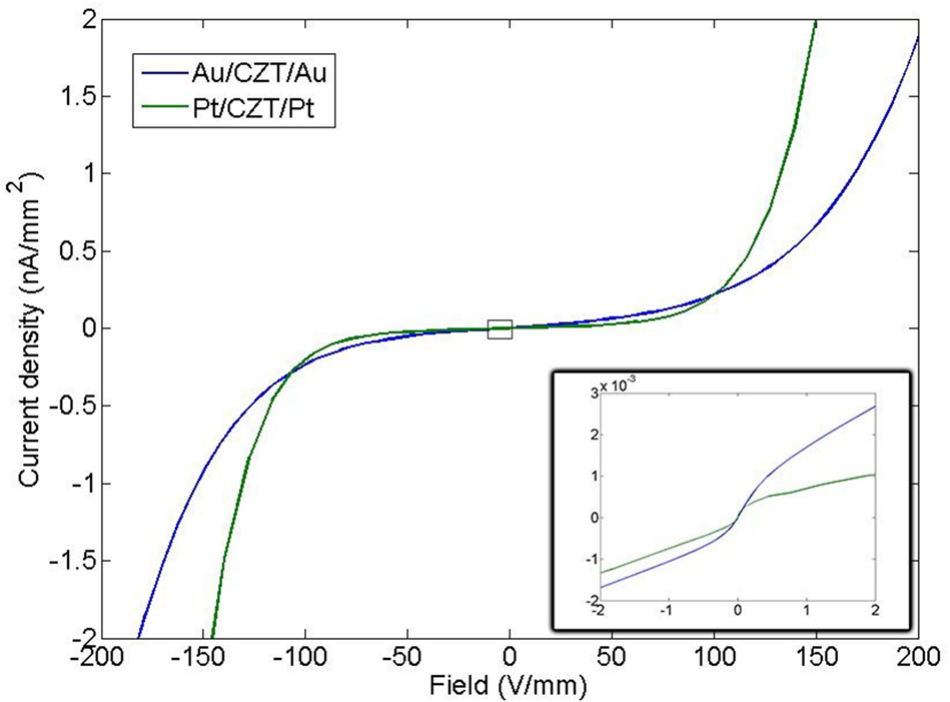
JV comparison for AA and PP samples. In the small pane are shown the JV between ± 2 V/mm.

**Figure 5:**
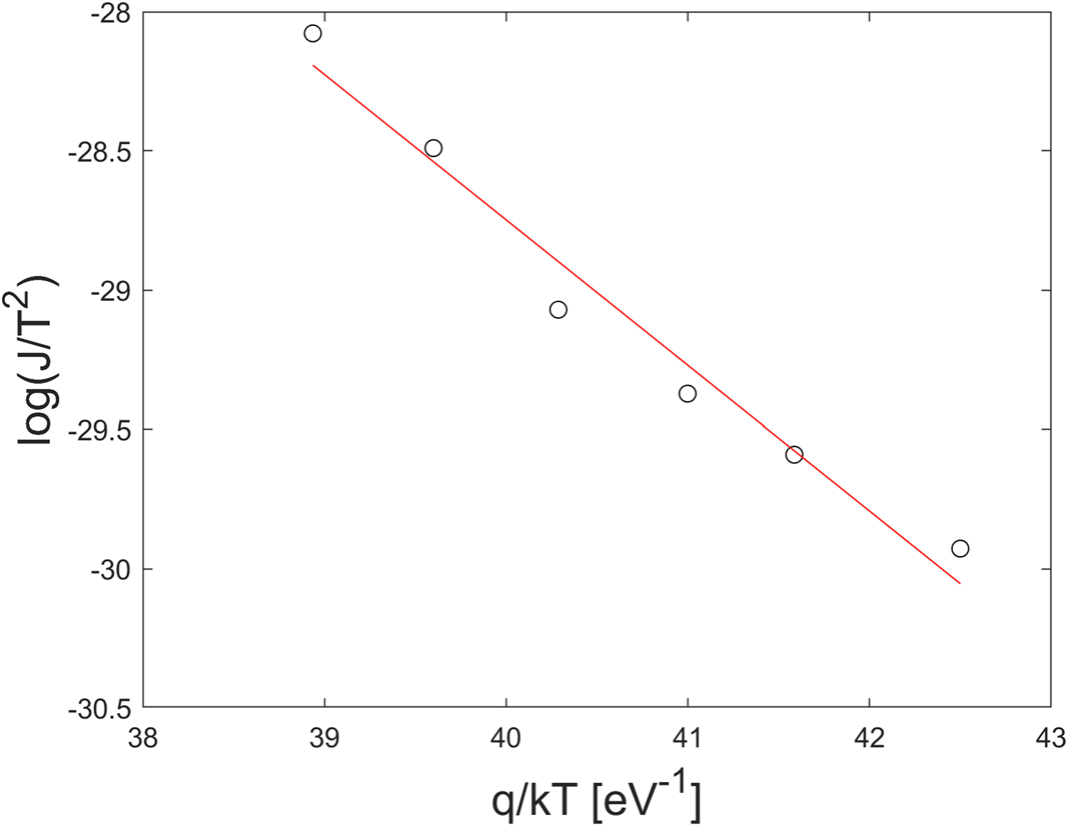
Arrhenius plots $log\left(J/{T}^{2}\right)$ versus $\frac{q}{kT}$ for the Pt detector.

**Figure 6:**
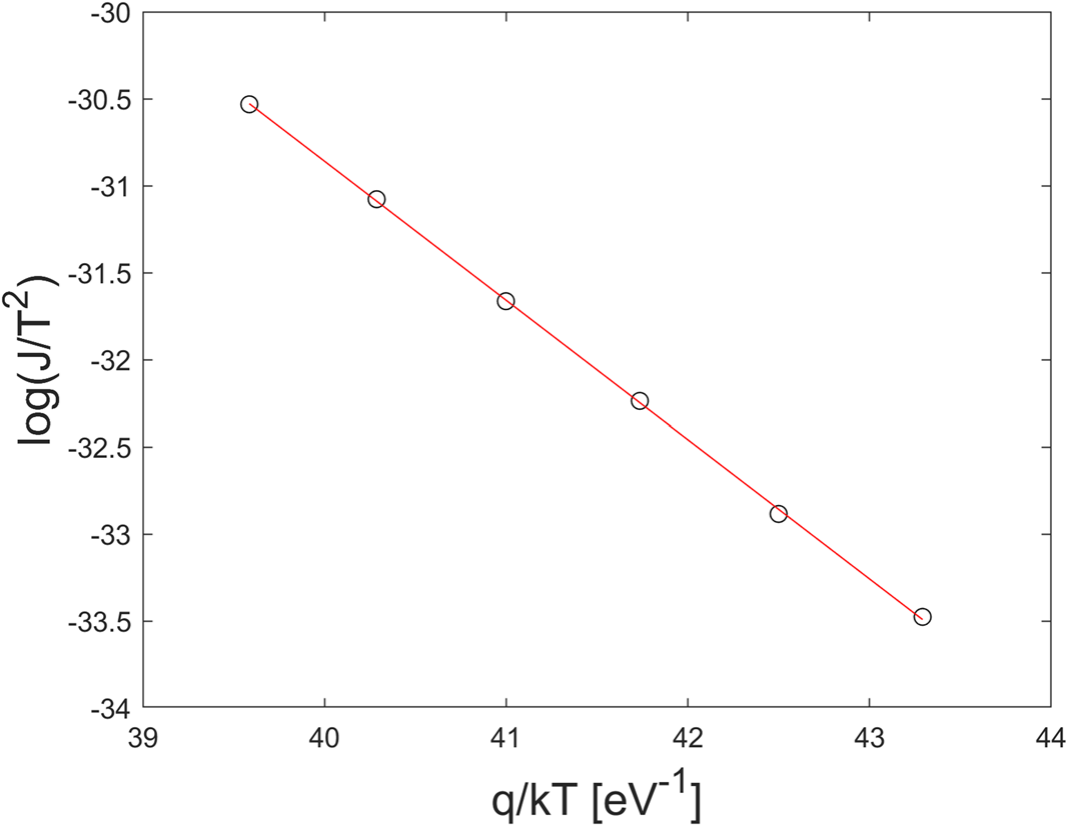
Arrhenius plots $log\left(J/{T}^{2}\right)$ versus $\frac{q}{kT}$ for the Au detector.

The current- voltage characteristics of the Pt and Au detectors are shown in Fig. 4. At low voltages (the inset of Fig. 4), both samples highlight a “S” shape curve, typical of metal–semiconductor–metal (MSM) contacts with two back-to-back Schottky barriers^24–26^. In this region, the current is dominated by the bulk resistivity and the contact resistance. The Pt detector shows a lower current and a better ohmic contact than those of Au detector, confirming the results reported in^27^. At high voltages, an exponential behaviour is observed: the crystal is fully depleted and the transport mechanism is governed by the interfacial layer–thermionic-diffusion (ITD) model^4^. This model foresees the existence of a very thin insulating layer, probably formed during the contact deposition, between the contact and the semiconductor material. This oxide layer can be due to the oxidation of Te-rich layer of CZT surface before Pt deposition^28^. ITD model incorporates the thermionic-diffusion theory across the Schottky barrier between the metal on the lightly doped semiconductor with the tunneling transport mechanism across the thin oxide layer^29^. According to the ITD model, the CZT detector with negative voltage at the cathode can be treated as a metal–semiconductor system consisting of a reversed-biased Schottky barrier at the cathode^4^. In this case the reverse current J is due to the majority carriers of the CZT crystal. For n-type CZT and in the voltage range where the thermionic-emission dominates J can be expressed as^30^:

1 $$J={\theta }_{n} {A}_{n}^{*}{T}^{2}{e}^{-\frac{{\phi }_{B0}}{{V}_{TH}}}{e}^{\frac{{VC}_{2}}{{V}_{TH}}}$$

where $V$ is the reverse voltage on the CZT/Metal junction, ${V}_{TH}=kT/q$ is the thermal voltage, ${A}_{n}^{*}=120 \;{m}_{e}^{*}/{m}_{e}$ is the is the electron effective Richardson constant, where ${m}_{e}^{*}$ and ${m}_{e}$ are the effective and free electron mass, respectively. ${\phi }_{B0}$ is the energy barrier height under thermal equilibrium conditions, ${\theta }_{n}$ , with $0<{\theta }_{n}<1$, is the electron transmission coefficient across the interfacial layer and ${C}_{2}=\frac{{\varepsilon }_{i}}{{\varepsilon }_{i}+2{q}^{2}\delta {D}_{s}}$ where ${\varepsilon }_{i}$ and δ are the permittivity and thickness of the interfacial layer, and ${D}_{s}$ is the density of surface states per unit energy and area. For p-type CZT material, Equation holds by replacing ${\theta }_{n}$ and ${A}_{n}^{*}$ with the hole transmission coefficient ${\theta }_{h}$ and the hole effective Richardson constant ${A}_{h}^{*}=120 {m}_{h}^{*}/{m}_{e}$, where ${m}_{h}^{*}$ is the effective hole mass. For CdTe crystals, ${m}_{e}^{*}=$ 0.1 and ${m}_{h}^{*}=0.7$^26^. By assuming n-type CZT material and following the procedure used in^30^ we estimated ${\phi }_{B0}$, ${\theta }_{n}$ and ${C}_{2}$ for both samples, by measuring the reverse *J-V* curves at different temperatures (− 5 °C; 25 °C). In particular, ${C}_{2}$ was obtained by fitting (Eq. 1) the reverse J–V curves in the high voltage region at different temperatures. In the voltage range where the coefficient ${C}_{2}$ is independent of temperature the thermoionic-emission dominates. The values of ${\phi }_{B0}$ and ${\theta }_{n}$ have been extracted from the Arrhenius plots $log\left(J/{T}^{2}\right)$ versus $\frac{q}{kT}$, as shown in Figs. 5 and 6 for the Pt and Au detectors at V = − 150 V, respectively. The values of the parameters are shown in Table 1. Both samples show similar values of the barrier height and the transmission coefficient, in agreement with the literature^4,30,31^. While, the coefficient ${C}_{2}$ of the Pt contact is larger than the Au one. This explains the prompt increasing of the current of the Pt detector at voltages above 100 V/mm. ${C}_{2}$ depends on the product $\delta {D}_{s}$, i.e. on the thickness of the oxide layer and the density of surface states at the CZT/metal interface. The transmission coefficient is related to the thickness of the oxide layer ( ${\theta }_{n}\propto {e}^{-\delta }$)^29^ and being the ${\theta }_{n}$ values of the Pt and Au contacts comparable, we can conclude that the higher leakage current in the Pt detector is due to the lower density of interface states of the Pt contact.

**Table 1:** Values of ${\phi }_{B0}$, ${\theta }_{n}$ and ${C}_{2}$ parameters extracted from the ITD model for the Pt and Au CZT detectors.

	${\phi }_{B0}$ (eV)	${\theta }_{n}$	${C}_{2}$
Pt	0.78 ± 0.03	0.05 ± 0.01	$1.56\times {10}^{-4}$
Au	0.75 ± 0.01	0.013 ± 0.005	$5.62\times {10}^{-5}$

### Spectroscopic performance

**Figure 7:**
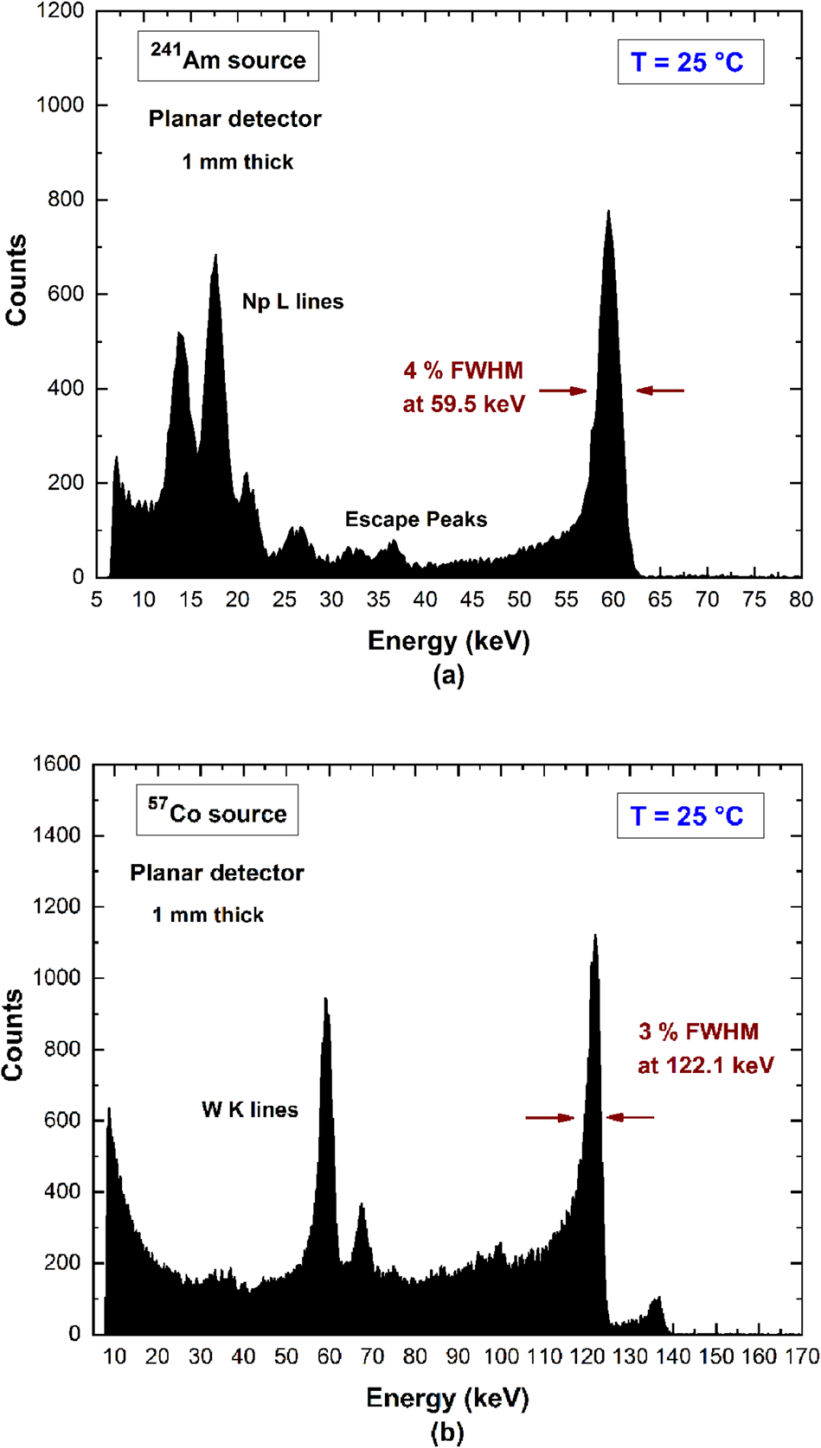
(**a**) Measured ^241^Am and (**b**) ^57^Co spectra with the Pt-CZT planar detectors.

The spectroscopic capabilities of the detectors were investigated. The measured ^241^Am and ^57^Co energy spectra at low rate conditions (< 300 counts/s) are shown in Fig. 7. The detectors were biased with an electric field of 1,500 V/cm at room temperature. The spectroscopic performances are comparable to Au-detectors, as previously presented in our works^32^. The effects of hole trapping (peak asymmetry and tailing) are clearly visible in the measured spectra, mainly in the ^57^Co energy spectrum. As widely documented in the literature^33,34^, both electrons and holes contribute to the formation of the charge pulses in planar detectors. In particular, photon interactions near the anode electrode create charge pulses with an increased hole contribute and, therefore, characterized by higher hole trapping effects. In our case, this mainly happens for the 122 keV photons of the ^57^Co source that are characterized by a mean free path of 1.9 mm on CZT material^35^. Generally, these effects can be mitigated by using unipolar detectors^33,34^, which charge pulses are mainly sensitive to the electrons. These detectors are typically realized with pixel or strip structures on the anode electrode^36,37^ and by using bi-parametric correction techniques^38,39^.

### Laser induced transient current technique

**Figure 8:**
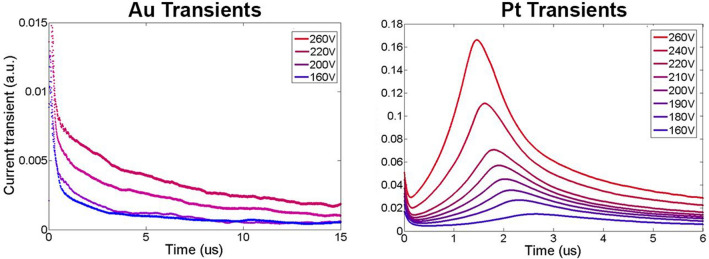
Typical current transients related to hole signal for Au (left) and Pt (right) contacts at different applied bias voltage.

LI-TCT demonstrated to be a powerful method to measure carrier transport properties^15,40–42^. However, in the papers published up to now, only electron transport properties were determined. This is because, the common gold contacts show a weak and noisy hole signal. On the other hand, we found out that samples with platinum contacts revealed a detectable hole signal. In Fig. 8, typical current transients for Pt and Au contacts, related to hole signal, are reported as example.

**Figure 9:**
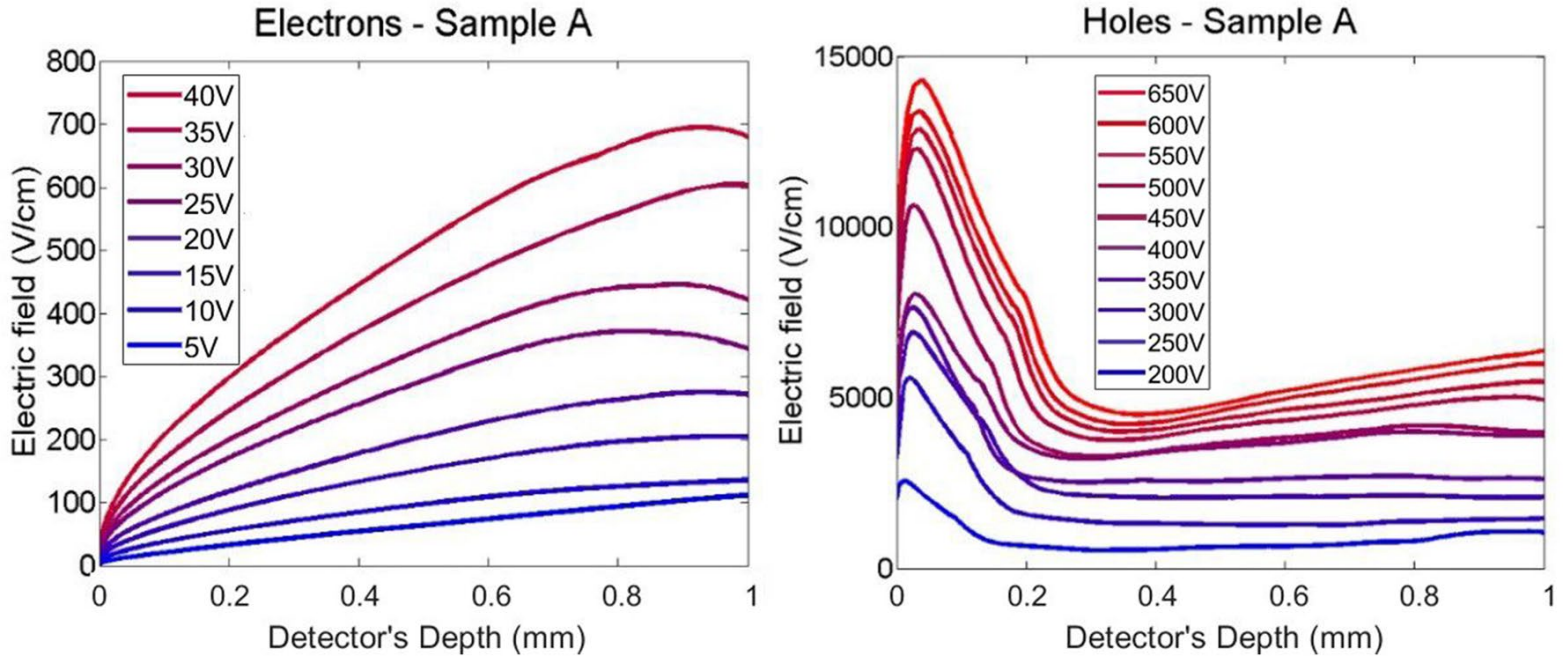
Electric field profiles for electrons (left) and for holes (right) of sample A at different bias voltage.

**Figure 10:**
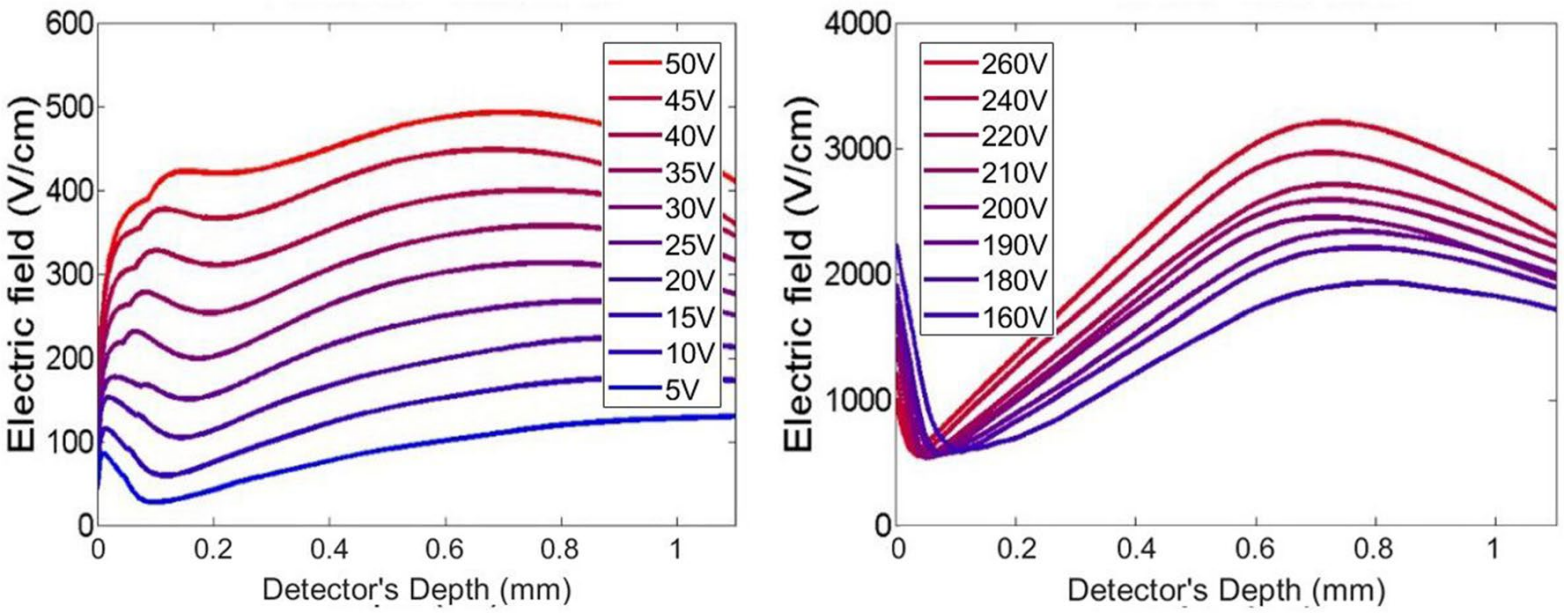
Electric field profiles for electrons (left) and for holes (right) of sample B at different bias voltage.

Figures 9 and 10 report the calculated electric field profiles of the two samples for both electrons and holes, as extracted analysing electrical transients as reported in^15^. It is advisable to underline that the calculated electric field profiles are those experienced by the charge carrier during the motion along the device and, as a consequence of different biases used for electrons and holes, a comparison between results for charge carriers of opposite sign is not so easy to do. In addition, the reconstruction of the electric field suffers from artefacts in proximity of the electrodes, especially near the illuminated one (on the left in Figs. 9 and 10). Since the profile of the field has been deduced from the current transient curves, the electric field near this electrode could be affected by the effect of a non-instantaneous light excitation that is not completely removed from the laser pulse deconvolution. Further, we cannot exclude the presence of space charge related to the interfacial defectiveness of the material.

The electric field calculated from LI-TCT transients for electrons shows in both samples a nearly linear increase that could be ascribed to an almost uniform negative space charge inside the sample. The higher slope in the sample A suggests a higher density of such space charge. Trapping of negative charge is realistic because the carriers travelling along the device are electrons.

Electric field reconstructed from hole transients exhibits a strong decreasing slope in the first 100 ÷ 200 microns close to the illuminated electrode. This phenomenon is stronger in sample A and it can newly traced back to trapping of negative charges localized mainly under the anode. When the distance from the cathode is greater than about 100 ÷ 200 μm from the illuminated electrode, sample A shows an almost uniform trend for the electric field whereas the trend in the sample B is growing up to about 600 μs it begins to decrease. The difference between sample A and sample B could be justified taking into account the residual negative charge trapping, already proved by electron measurements, that is now compensated by holes trapping along the thickness of detectors. The effect of compensation is less effective in sample A due to both a higher negative space charge density and a higher hole mean free path with respect sample B, in which a relative maximum of electric field is reached at about 600 μs in depth.

Mean free path for electrons and holes can be calculated by the transport parameters obtained for both carriers and reported in Table 2. All results are in agreement with literature values reported for CZT^15,43,44^.

**Table 2:** Carriers mobility and lifetime extracted from LI-TCT measurements.

Sample	Electrons mobility ${{{\mu}}}_{{{e}}}$ (cm^2^/Vs)	Electrons life-time ${{{\tau}}}_{{{e}}}$(μs)	Holes mobility ${{{\mu}}}_{{{h}}}$ (cm^2^/Vs)	Holes life-time ${{{\tau}}}_{{{h}}}$(μs)
A	1,408	0.65	36	1.97
B	1,032	0.65	28	> 5

The values of hole mobility in the two samples are similar as expected. The model is unable to provide the holes lifetime for sample B since times of flight is shorter than lifetime for all the investigated applied voltages. Because of this, we have not enough data to reconstruct the carrier trapping process. The only way to increase time of flight is to decrease the bias voltage. However, measuring transients with voltages lower than 100 V is not trivial since the signal becomes comparable with noise. We can only assume that the lifetime is greater than the highest time of flight (5 μs for sample B). Sample A shows a significant lower hole lifetime: such difference can be easily ascribed to the different growth technologies.

## Discussion

We realized Pt Schottky contacts deposited on CdZnTe with a novel procedure based on the electroless technique. The use of methanol/DMF as solvent allows the formation of homogeneous and mechanically stable contacts, able to withstand a standard tape test.

TEM analysis showed that a continuous Pt layer is formed in contrast to water deposition. Below the Pt layer, a PtTe_2_ alloy is formed. This is a different situation with respect to gold electroless deposition, where a Te layer is formed as a consequence of Cd depletion process^8,19^.

Electrical characterisation showed that the Pt contact in the ± 2 V/mm range has lower current density than that of Au contact. Probably, this is due the higher energy barrier of platinum contacts (see Table 1). At high voltages, the current density of platinum contacts increases and exceeds that of gold contacts. According to the ITD model, this behaviour can be explained by the lower density of states at the metal semiconductor interface, probably related to the formation of the PtTe_2_ alloy.

Energy resolution of γ-ray spectra for detectors realized with Pt contacts is comparable with those realized using gold contacts so that platinum is a good contact for spectroscopic applications.

Thanks to platinum contacts, for the first time we were able to measure hole mobility and lifetime by means of LI-TCT, while, with gold contacts, that was not possible. This also means that Pt contacts actually ease the hole collection with respect to gold contacts. The admirable hole collection efficiency of Pt/CZT contacts can be exploited for detectors working under high flux conditions, where the removal of low mobility holes is generally an issue^45^.

Further studies are ongoing in order to deposit a thicker electroless Pt layer without the need of sample heating. Room-temperature deposition would ease the photolithographic process and open the possibility to pattern fine-pitch electrodes.

## Supplementary information

Supplementary Information 1.
