# Supplementary material for: Improved electroless platinum contacts on CdZnTe X- and γ-rays detectors
Source: Sci Rep. 2020 Aug 13;10:13762. doi: 10.1038/s41598-020-70801-9 (PMC7426932; doi:10.1038/s41598-020-70801-9)
Supplement: Supplementary file 1 — Supplementary Information 1. [file 41598_2020_70801_MOESM1_ESM.docx]

# Supplementary information


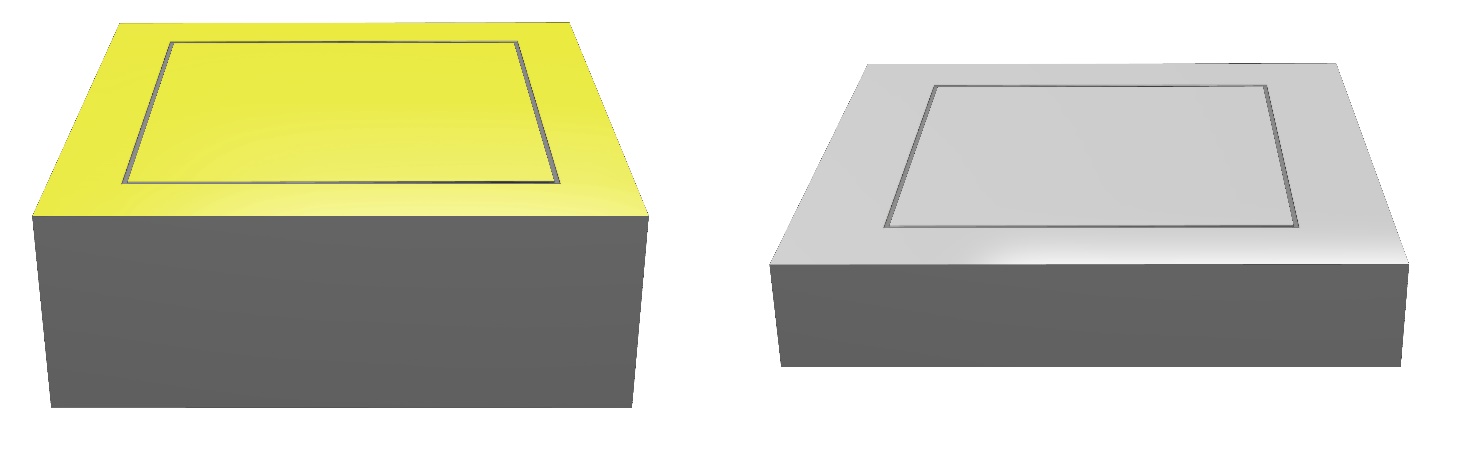


Figure S1. 3D models of the samples AA (left) and PP (right) used for I/V measurements and spectroscopic characterization.


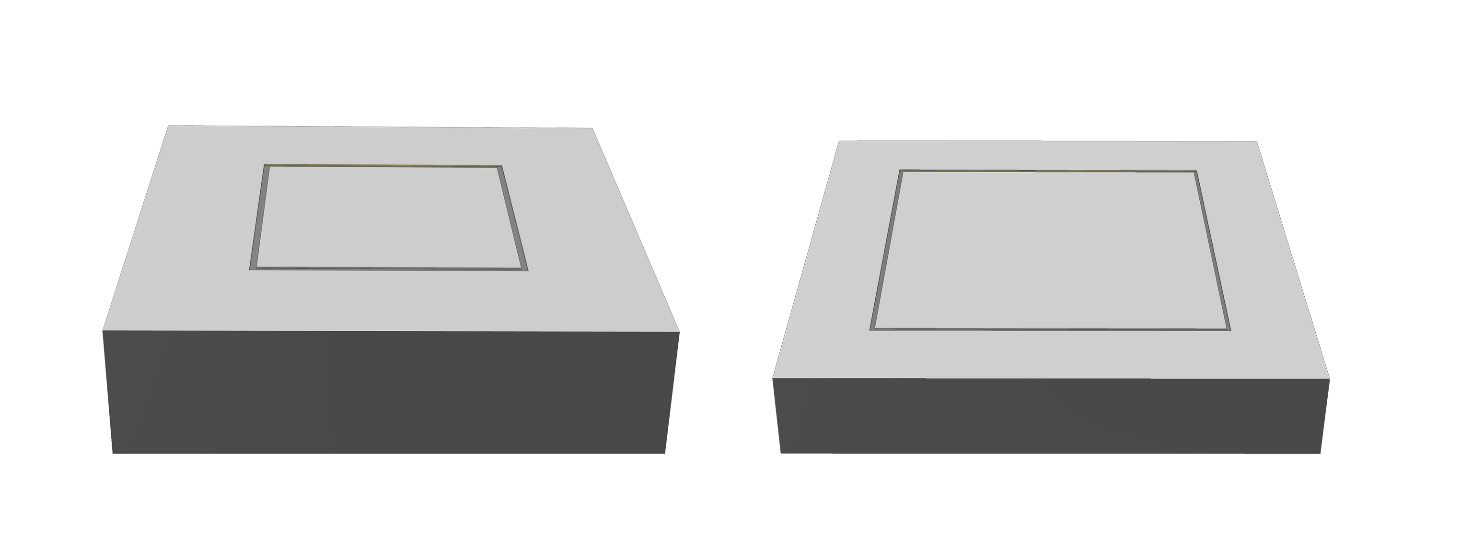


Figure S2. 3D models of the samples A (left) and B (right) used for laser induced transient current technique characterization.
